# Supplementary material for: Marine biofilms: cyanobacteria factories for the global oceans
Source: mSystems. 2024 Oct 15;9(11):e00317-24. doi: 10.1128/msystems.00317-24 (PMC11575276; doi:10.1128/msystems.00317-24)
Supplement: Supporting information — Fig. S1-S18, Tables S1 and S2, and additional experimental details. [file msystems.00317-24-s0001.docx]

Marine biofilms: cyanobacteria factories for the global oceans

Cheng Zhong ^†,‡^, Shun Yamanouchi ^§^, Yingdong Li ^†,‡^,Jiawei Chen ^†,‡^, Wei Tong ^†,‡^, Ruojun Wang ^†,‡^, Kun Zhou ^†,‡^, Aifang Cheng ^†,‡^, Weiduo Hao ^||^, Hongbin Liu ^†,‡^, Kurt O. Konhauser ^||^, Wataru Iwasaki ^§,¶^, and Pei-Yuan Qian ^†,‡ *^

**Affiliations**

† Department of Ocean Science, The Hong Kong University of Science and Technology, Hong Kong, China.

‡ Southern Marine Science and Engineering Guangdong Laboratory (Guangzhou), Guangzhou, China.

§ Department of Biological Sciences, Graduate School of Science, the University of Tokyo. Bunkyo-ku, Tokyo, 113-0032, Japan.

|| Department of Earth and Atmospheric Sciences, Faculty of Science, University of Alberta, Edmonton, Alberta, T6G 2E3, Canada.

¶ Department of Integrated Biosciences, Graduate School of Frontier Sciences, the University of Tokyo, Kashiwa, Chiba, 277-0882, Japan.

* Corresponding author. Email: boqianpy@ust.hk

Cheng Zhong: chengzhong@swpu.edu.cn

Shun Yamanouchi: [yamanouchi@bs.s.u-tokyo.ac.jp](mailto:yamanouchi@bs.s.u-tokyo.ac.jp)

Yingdong Li: [ylifc@connect.ust.hk](mailto:ylifc@connect.ust.hk)

Jiawei Chen: [jchenek@connect.ust.hk](mailto:jchenek@connect.ust.hk)

Tong Wei: tweiac@connect.ust.hk

Ruojun Wang: [rwangaw@connect.ust.hk](mailto:rwangaw@connect.ust.hk)

Kun Zhou: [zhoukk@ust.hk](mailto:zhoukk@ust.hk)

Aifang Cheng: [chengaf@ust.hk](mailto:chengaf@ust.hk)

Weiduo Hao: [whao@ualberta.ca](mailto:whao@ualberta.ca)

Hongbin Liu: [liuhb@ust.hk](mailto:liuhb@ust.hk)

Kurt O. Konhauser: [kurtk@ualberta.ca](mailto:kurtk@ualberta.ca)

Wataru Iwasaki: iwasaki@k.u-tokyo.ac.jp

Supplementary Text

**Alignment and Bayesian tree inference**

The metagenome-derived 16S rRNAs were first clustered by 97% identity by using CD-HIT v.4.8.0 [1] to remove redundancy, resulting in 43 clusters. Preliminary analysis revealed that one cluster (represented by the sequence pf_W-HK1707.PFspades_42) caused a long branch attraction, rendering the tree root ambiguous. Thus, it was removed, and the representative sequences of the resulting 42 clusters were subjected to subsequent analyses.

A non-clock molecular phylogenetic tree was then reconstructed to infer cyanobacterial 16S rRNA phylogenetic relationships (i.e., tree topology). Following Schirrmeister et al. [2], 16S rRNA sequences from known cyanobacterial sequences were added to our dataset to confirm the consistency of our results with those of previous studies and obtain calibration points for subsequent clock analysis. 16S rRNA gene extraction/examination of the total 58 sequence records, which represent either of 16S rRNAs, rRNA operons or complete genomes, was performed using Barrnap v.0.9 [3] to obtain standardised and comparative 16S rRNA genes. As a result, 56 cyanobacterial 16S rRNA sequences were retained from the 58 cyanobacterial genomes. Furthermore, three non-cyanobacterial species (Beggiatoa sp., Chlamydia trachomatis and Spirochaeta thermophila) were added as outgroup species. Their rRNA sequences were retrieved from the NCBI GenBank database (accession numbers NR_041726.1, NR_025888.1 and NR_117123.1, respectively). All of these sequences were retrieved from the NCBI GenBank database, and the accession numbers can be found in Schirrmeister et al. [2].

Subsequently, 101 unique 16S rRNA sequences (42 metagenome-derived sequences, 56 references and 3 outgroup species) were aligned using MAFFT v.7.480 with --globalpair--maxiterate 1,000 options (a slow but an accurate strategy) [4]. Sequence trimming was not performed to retain as many evolutionarily informative columns as possible. The GTR+I+G4 model was proposed to be the best fit for the multiple sequence alignments, according to Modeltest-NG v.0.1.6 [5]. Phylogenetic relationships were reconstructed in Bayesian inference framework by using MrBayes v.3.2.7a [6]. Forty independent Markov Chain Monte Carlo (MCMC) runs were performed, each of which computed four Metropolis-coupled chains for 5,000,000 generations with a sampling frequency of 500. As many as 23 MCMC runs out of the 40 runs did not mix well and failed to converge. Thus, a consensus phylogenetic tree was constructed using the remaining 17 runs. The 17 runs were visually confirmed to have mixed well and converged using Tracer v.1.7.2 [7]. The effective sample size for all parameters was >10,000. A conservative burn-in was performed by dropping the first 25% of the samples to ensure that they were from a stationary phase. The tree samples were summarised by the ‘sumt’ command of MrBayes as the 50%-majority rule consensus tree.

Bayesian molecular clock analysis was conducted using BEAST v.2.6.3. Input XML files for the BEAST software were generated using BEAUti v2.6.5, a graphical user interface included in the BEAST package. We set monophyletic constraints and calibration points according to Schirrmeister et al. (see the next section), as well as the GTR+I+G4 substitution model and the uncorrelated log-normal distribution of clock rates.

While performing Bayesian model estimation with MCMC, the BEAST program halted because the initial random trees did not satisfy our constraints. Thus, we performed a ‘preliminary calibration’ under moderate constraints, and then a ‘recalibration’ under the original conditions using the phylogenetic tree obtained in the preliminary calibration as a starting tree. In the ‘preliminary calibration’, the constraint on the tree root age was relaxed and a Gaussian prior distribution with a mean of 3 and a standard deviation of 2 was assumed. A single MCMC run was performed for 10,000,000 generations with a sampling frequency of 1,000. After dropping the first 25% samples, the maximum clade credibility tree was obtained using TreeAnnotator v.2.6.3. The tree was ‘recalibarated’ using the resulting consensus tree as the starting tree. Under the original constraints, 16 independent MCMC runs were performed for 100,000,000 generations with a sampling frequency of 1,000. The runs were visually confirmed to have mixed well and converged by using Tracer v.1.7.2. The effective sample size for all parameters was > 480. The run with the best average of the likelihoods of least 75% was adopted, and the run was summarised with TreeAnnotator to obtain the final maximum clade credibility tree.

**Phylogenetic constraints and calibration points**

First, the age of the most recent common ancestor (MRCA) of cyanobacteria was assumed to be dated to before 2.45 Ga (the upper limit of the GOE) and after 3.8 Ga (the lower limit of the birth of life). Thus, the prior distribution of tree height was set to uniform distribution between 2.45 and 3.8. Second, Gloeobacter violaceus and Synecococcus sp. P1 were assumed as the most basal and the next basal species, respectively. Thus, two monophyletic constraints were imposed on the other species. Third, multicellular cyanobacteria were assumed to have shared ancestry, and their common ancestor had emerged certainly before 2.1 Ga (the earliest fossil record of terminally differentiated cyanobacteria) and most likely after 2.45 Ga (the upper limit of the GOE). These constraints were implemented by identifying the internal node representing the MRCA of multicellular cyanobacteria (referred to as ‘node 3’ in Schirrmeister et al. 2) in our non-clock tree. Then, a monophyletic constraint was imposed on the tips under node 3, and the prior distribution of the age of node 3 was set to a log-normal distribution with an offset of 2.1, a mean of 2.27 and a standard deviation of 0.5. (Note that ‘mean’ and ‘standard deviation’ refer to values in the real space.) Finally, cyanobacteria with their terminal cells differentiated were assumed to have shared ancestry, and their common ancestor emerged before 2.1 Ga and after 2.45 Ga for the same reason as multicellular cyanobacteria. Likewise, the node of their common ancestor (referred to as ‘node 31/32’ in Schirrmeister et al. 9) was identified from the non-clock phylogenetic tree, monophyletic constraints were imposed on its descendants and the age prior of node 31/32 was set to a log-normal distribution with an offset of 2.1, a mean of 2.23 and a standard deviation of 1.0. Note that these parameter settings were based on the BEAST input XML file (Dataset_S02) attached as a supporting information by Schirrmeister et al. 2, and some of them were different from the description in their article.

**Estimation of linage-specific diversification rates**

The following (hyper) prior distributions were assumed. First, the prior distribution of the speciation rates was set to the discretised log-normal distribution with six categories. The mean parameter of the log-normal distribution was assumed to be log-normally distributed between 10−6 and 102. The standard deviation parameter was modelled as the exponential distribution with a mean of 0.587405 to ensure that the log-normal distribution was expected to cover exactly one order of magnitude with 95% prior probability. Second, the extinction rates were assumed to be constant for all categories, and they were sampled from a log-uniform distribution between 10−6 and 102. Third, the rate parameter for rate-shift events was assumed to be uniformly distributed between 0 and 100 / (total branch length) to express the belief that the expected number of rate-shift events was 100 at most. The transition rate matrix between categories was set to the Jukes–Cantor-like model (i.e., all substitution rates were presumed to be equal, scaled by the event rate parameter). Finally, incomplete taxon sampling was accounted for by setting the sampling parameter to 73/141. This setting was adopted because our phylogenetic tree contained 73 tips (sequence clusters with 99% identity) and our metagenome-derived 16S rRNA sequence dataset contained 141 unique sequences (sequence clusters with 100% identity). The initial values of the parameters of speciation and extinction rates were set to (number of tree tips − 2) / (total branch length) and half of that, respectively. Four independent MCMC runs were performed for 250,000 generations with a sampling frequency of 500 and a tuning interval of 200. The resulting traces were mixed and combined in the settings of the RevBayes ‘mcmc’ function. The runs were visually confirmed to have properly converged with Tracer v.1.7.2. The effective sample sizes for all parameters were >800. The first 25% of the combined trace was discarded as burn-in, and linage-specific net diversification rates (i.e., speciation rates minus extinction rates) were visualised and summarised as the median values of the MCMC samples by using the R packages ggtree v.3.0.4 [8] and RevGadgets v.1.0.0. [9]. The estimation results, especially the disparities in net speciation rates among lineages, were ensured to not have been affected by the prior distribution by also performing the above analysis but under different settings in terms of the number of rate categories (3 and 12), the mean parameter of the standard deviation parameter’s hyperprior distribution (0.587405 × 2 and 0.587405 × 3) and the sampling parameter (73/(73 × 8) and 73/(73 × 32)).

**Taxonomic classification and functional annotation of metagenome-assembled genomes and reference genomes**

The custom reference datasets for functional analyses contained 69 cyanobacterial full-genome sequences with an additional 63 *Synechococcus* and 13 *Prochlorococcus* full-genome sequences (maximum available) selected from GenBank. Most of 69 cyanobacterial genomes were chosen from a previous study to best represent phylogenetic disparity [10], and they could cover well the biofilm-forming (16S rRNA genes) taxonomic clades or relatives.

The phylogenomic tree of the 69 full-genome sequences was reconstructed using GToTree v.1.6.11 on the basis of cyanobacteria single-copy gene set (251 gene targets). After confirming the consistency of the phylogenomic tree with that of a previous study [10], the phylogenomic tree of these 148 compiled MAGs/genomes (10 biofilm-forming MAGs, 62 non-seawater predominant genomes, 63 *Synechococcus* genomes, and 13 *Prochlorococcus* genomes) was reconstructed following the same method because of the importance of the planktonic cyanobacterial lineages as revealed by the 16S rRNA-based phylogenetic tree. The 62 non-seawater predominant genomes were obtained after de-replication of 69 full genomes with the 63 *Synechococcus* genomes, and 13 *Prochlorococcus* genomes and their taxa were covered by the biofilm-derived cyanobacterial lineages.

**Results and discussion**

**Taxonomic profiling and Venn diagram analysis**

For seawater, *Synechococcus* (average 49.4% of the total cyanobacteria) and *Prochlorococcus* (average 39.1% of the total cyanobacteria). Changes in microbial genera and ocean regions were more profound in the marine biofilm samples than in the seawater (the relative abundance of the top 50 genera in the 101 marine biofilm and 91 seawater samples are shown in Fig. S5). For marine biofilms, top five genera are *Synechococcus* (average 26.0% of total cyanobacteria), *Pleurocapsa* (average 12.1% of total cyanobacteria), Unclassified cyanobacteria (9.57%), *LyngGa* (average 7.5% of total cyanobacteria), and *Phormidium* (average 5.4% of total cyanobacteria). Combined with taxonomic profiling and Venn diagram analyses, we identify Nostocophycideae and Oscillatoriophycideae are only two cyanobacteria subclasses contain biofilms-specific memberships. Within the two subclasses, mostly Chroococcidiopsis, followed by Oscillatoria spongeliae and unclassified genera of Cyanothece, Crinalium, unclassified Nostocaceae_Group, and unclassified Cyanobacterium are detected.

The Oscillatoriophycideae was also dominant in modern microbial mats and their lithified equivalents, i.e., stromatolites [11,12]. Using the relative abundance of Oscillatoriophycideae as markers, the niche differentiation intensity for biofilms on rocks (82.6% of total community) was two-fold higher than that for biofilms on other substrata.

In contrast, subclasses Sericytochromatia (ML635J_21), Nostocophycideae, and Synechococcophycideae contained memberships unique to seawater, including Cylindrospermopsis raciborskii, genera *LeptolyngGa*, *Synechococcus*, and unclassified *Sericytochromatia*. *Sericytochromatia* is nonphotosynthetic and was proposed to be the basal to oxygenic photosynthesis cyanobacteria [13].

Artificial panels are used in parallel to natural materials because sampling natural substrata in the subtidal zone with controllable developing time and recording its formation is technically challenging. We examine the taxonomic distance to natural medium and confirm the effects from sampling materials are negligible relative to the distance from marine biofilms to seawater. With regards to our focus on early Earth, a further substrata analysis shows that the main trend across both natural materials and rock substrata is consistent. While, rock substrata have a significantly (p < 0.05) higher relative abundance of cyanobacteria (26.5%) compared to other substrata (0.8% - 12%) and seawater (7%-10%), and the Oscillatoriophycideae subclass in rock substrata (82.6%) is 2-folds higher than the average of other substrata (40.4%).

**Cyanobacterial biogeography and the Red Sea**

Metagenomic samples were used as those were originally collected across multiple temporary and spatial intervals, including a total of 101 marine biofilms and 91 epipelagic seawater. Marine biofilm metagenomic data were originally developed from seawater from the South China Sea (including 7 rock samples), the East China Sea, the Red Sea, and the North Atlantic Ocean. Seawater was collected in the adjacent environments of the marine biofilms and additional 6 more oceanic regions.

Network analyses can identify complex co-occurrence patterns and relationships between microbial taxa and sampling locations. A tight cluster of the Red Sea samples based on biofilm-forming cyanobacteria community compositions was outstandingly different in the network. The seawater cyanobacteria community cluster in the Red Sea also shifted off seawater cyanobacteria from other marine regions.

To further investigate the cyanobacterial community structure in the Red Sea, we compared the taxonomic compositions and diversity in marine biofilms and seawater from the Red Sea with that from other marine regions (Fig. S14). Importantly, a unique cyanobacterial structure in the Red Sea was identified. For cyanobacteria normally below the detection limit in seawater (i.e., biofilm-forming cyanobacteria), the Red Sea cyanobacteria were more relatively abundant and diverse than other regions' general pattern, except for rock substrata samples.

The unique cyanobacterial pattern was also revealed by comparative cyanobacterial diversity and richness using Shannon and Chao1 indexes, respectively. Notably, seawater cyanobacterial diversity and richness were considerably lower than the other marine environments, including adjacent to the Indian Ocean and the Mediterranean Sea. In contrast, biofilm-forming cyanobacterial diversity and richness were generally higher than in the other studied marine regions.

We identified cyanobacteria lineages from the Red Sea samples that covered most of the phylogenetical clades. The Red Sea cyanobacterial lineages have high coverage for phylogenetic clades far from the planktonic cyanobacteria and close to the basal cyanobacteria groups. Whereases and cyanobacterial phylogenetic coverage for other marine regions were opposite. The divergence of the early lineages to the other subsection clade can date back to 2.5 billion years ago (Ga) at the Great Oxidation Event.

**Geochemical indicators of oxygenic photosynthesis before Great Oxidation Event**

The oxidation of Cr(III) in mafic rocks is likely due to the local O_2_ generation from cyanobacteria in benthic environments (e.g., terrestrial lands or shallow marine submerged areas) back to three billion years ago [14], which can mobilize Cr, and the isotopic fractionation in this process can be captured in shale formation and banded iron formation. Similarly, redox-sensitive metals such as U and Mo are highly soluble under local oxic conditions generated by benthic cyanobacteria, and such a local oxic environment can partially explain the enrichment of these metals in shales and banded iron formation [15].

**Chromatic acclimation**

Analysis of chromatic acclimation demonstrates that essentially new functional capacities that promote the geographical adaptation of cyanobacterial communities are also evolved. Lacking light utilisation is likely a major constraint for the dispersion of the biofilm-forming cyanobacteria in global oceans. The functional analysis showed that biofilm MAGs could only adsorb green light, which is a major light type in the shallow benthic environment and the euphotic layer of the pelagic realm [16]. In contrast, the seawater MAGs, consistent with *Synechococcus* and *Prochlorococcus*, contain genes for using blue light (pigment type 3c) and modifying their phycourobilin: phycoerythrobilin ratio (pigment type 3d) in response to changes in the colour of the ambient light through a process known as type IV chromatic acclimation in most of the pelagic realm [17,18].

Fig. S1 Substrata analysis showing the large variance between rock panels to the other materials; detailed comparison of cyanobacteria relative abundance among different substrata for biofilm development. Tara: sweater samples from *Tara* Oceans [19]. 0.1 µm: 24 seawater samples collected from Zhang et al. [20].

 Fig. S2 Heatmap showing the distribution of the relative abundance of top 50 cyanobacterial genera across biofilm and seawater from different ocean regions. The color and text codes in the upper part of the taxonomic profiles refer to the ocean names (see Fig. 1a for the full name of different ocean regions).


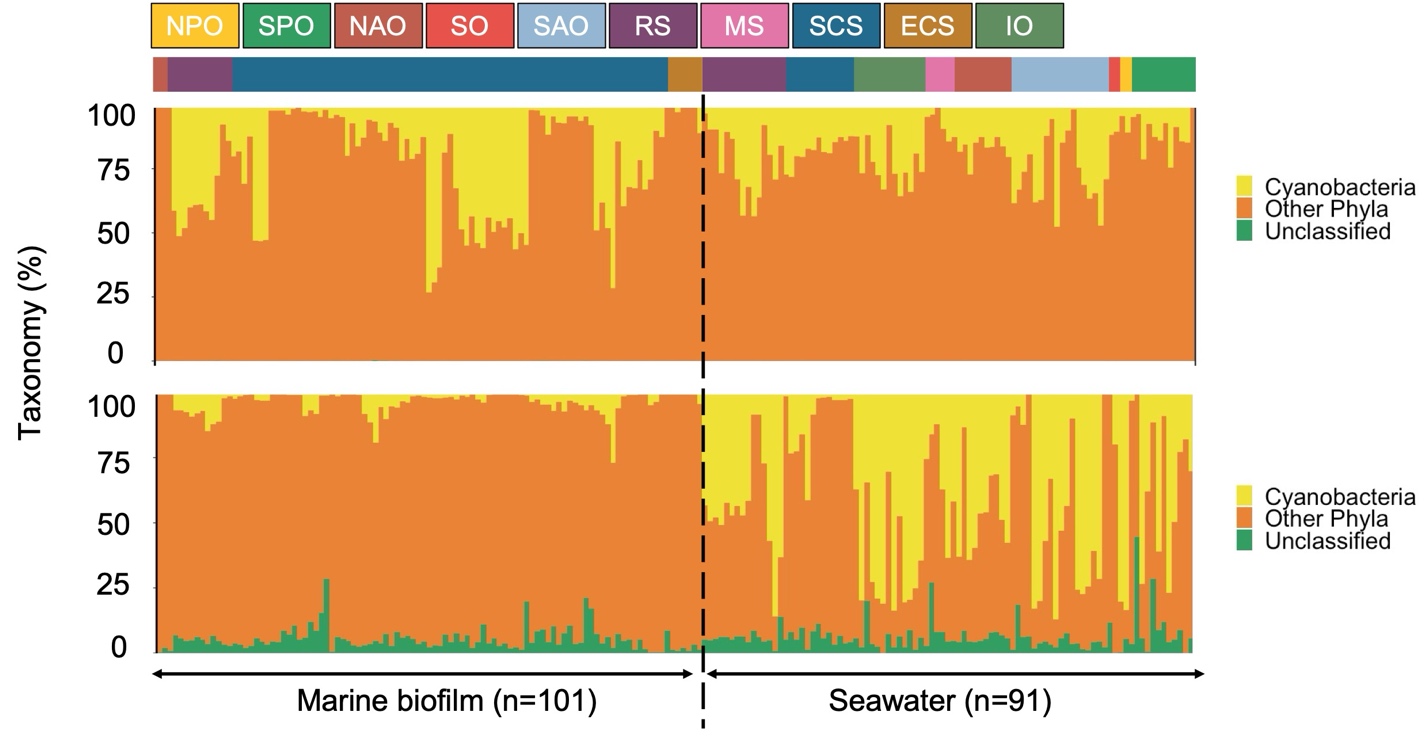


Fig. S3 Comparative taxonomic analysis of the cyanobacteria species based on 16S rRNA Illumina tags (upper panel) and protein marker genes (lower panel) at the phylum-level across marine biofilms and seawater. The color and text codes in the upper part of the taxonomic profiles refer to the ocean names (see Fig. S1 for the full name of different ocean regions).

Fig. S4 Subclass level bio-profiling PCoA. Cyanobacterial subclass Oscillatoriophycideae has different distribution compared to other classes such as Nostocophycideae and Synechococcophycideae.


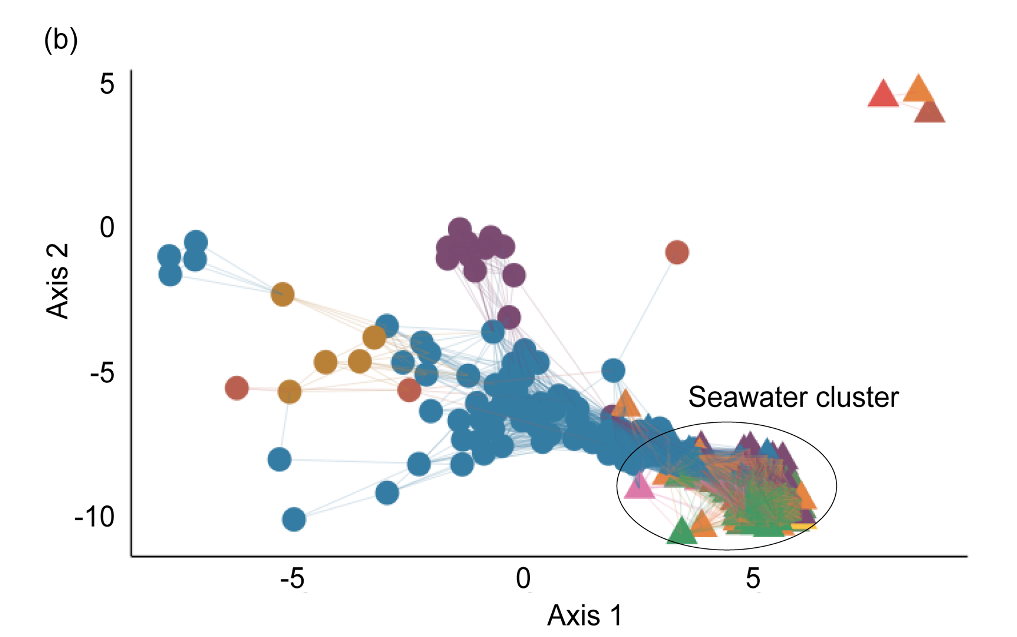


Fig. S5 Bray–Curtis distance network of biofilm and seawater samples across oceanic regions (the color of the regions refers to Fig. S1).

Fig. S6 Node degrees based on cyanobacteria community compositions for 101 biofilms and 91 seawater samples.

Fig. S7 Node betweenness centrality based on cyanobacteria community compositions for 101 biofilms and 91 seawater samples.

 Fig. S8 Node closeness centrality based on cyanobacteria community compositions for 101 biofilms and 91 seawater samples.

 Fig. S9 Node eigenvector centrality based on cyanobacteria community compositions for 101 biofilms and 91 seawater samples.

Fig. S10 Changes in cyanobacterial community dissimilarity (Bray–Curtis distance) with increasing geographical distance.

Fig. S11 Total co-presence of OTUs decreased with total sample number. Coloured dashed lines are linear regressions with R^2^ that are constructed for biofilm and seawater samples. The embedded plot shows the same analysis using a non-normalised dataset.

Fig. S12 (a) Seasonal changes in the relative abundance of cyanobacteria and (b) changes in the relative abundance of cyanobacteria as a function of development duration.

Fig. S13 Metagenomic patterns for samples collected from the Red Sea. (a) A family-level taxonomic classification of cyanobacteria, (b) network analysis and (c) diversity analysis across 101 marine biofilm and 91 seawater samples.


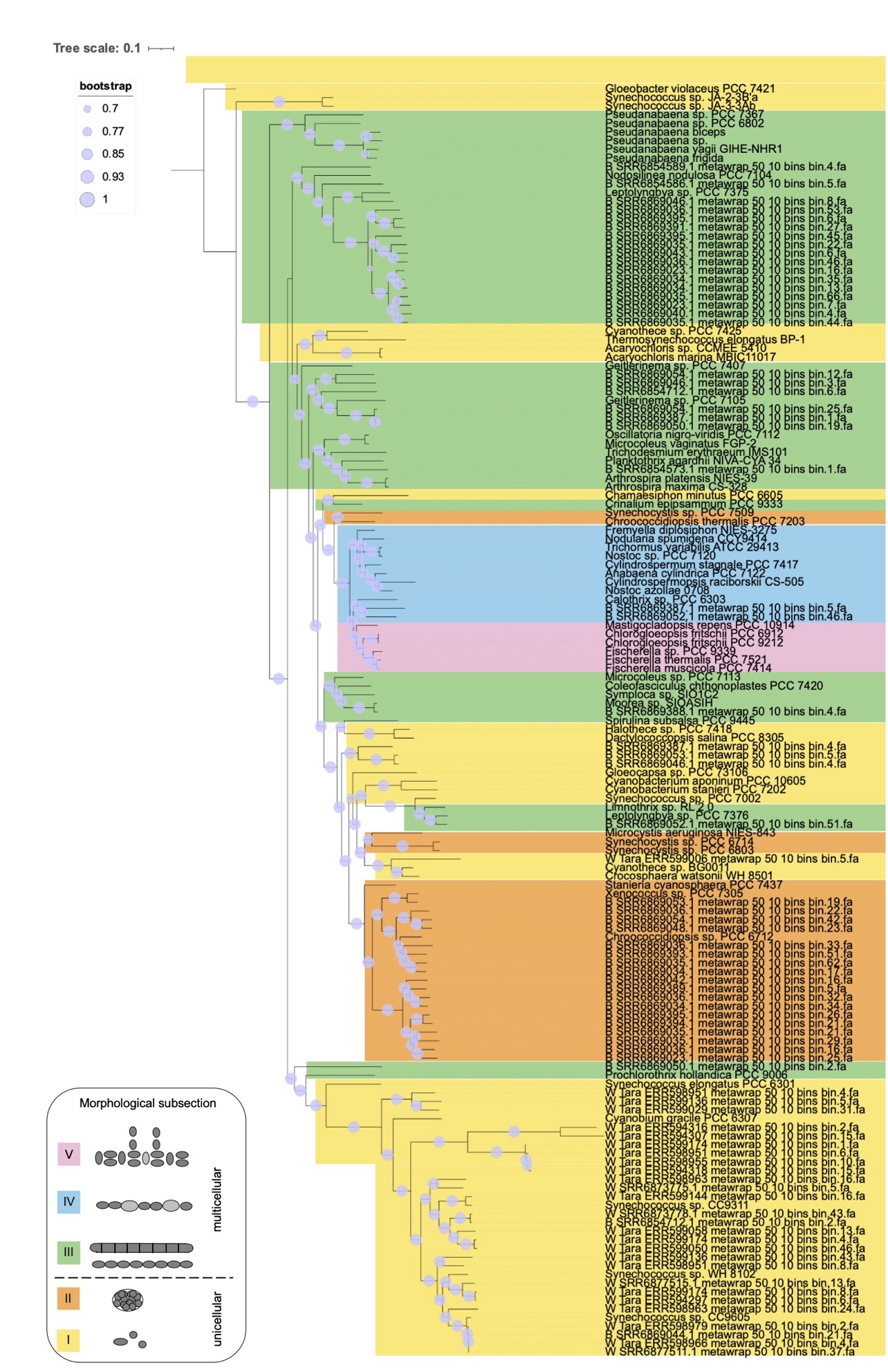


Fig. S14 Phylogenomic tree using metagenome-assembled genomes (MAGs) derived from biofilm and seawater samples, and reference genomes that represent each subsection of distinct morphology. The five subsections referred to Schirrmeister et al. [10].


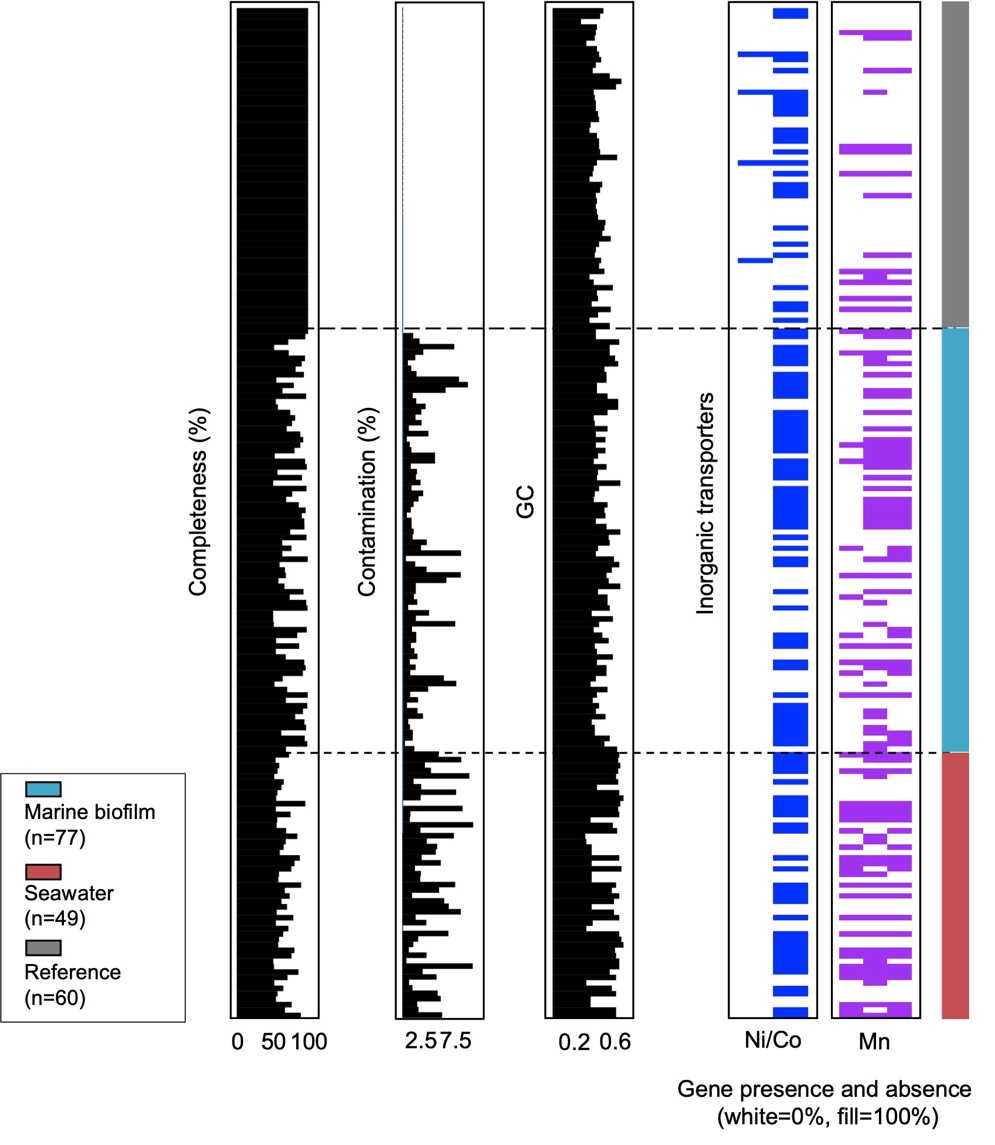


Fig. S15 Genome characteristics of 77 cyanobacterial MAGs from 101 biofilm samples and 49 cyanobacterial MAGs from 91 seawater samples, including completeness, contamination, GC content, Ni/Co and Mn Transporters.


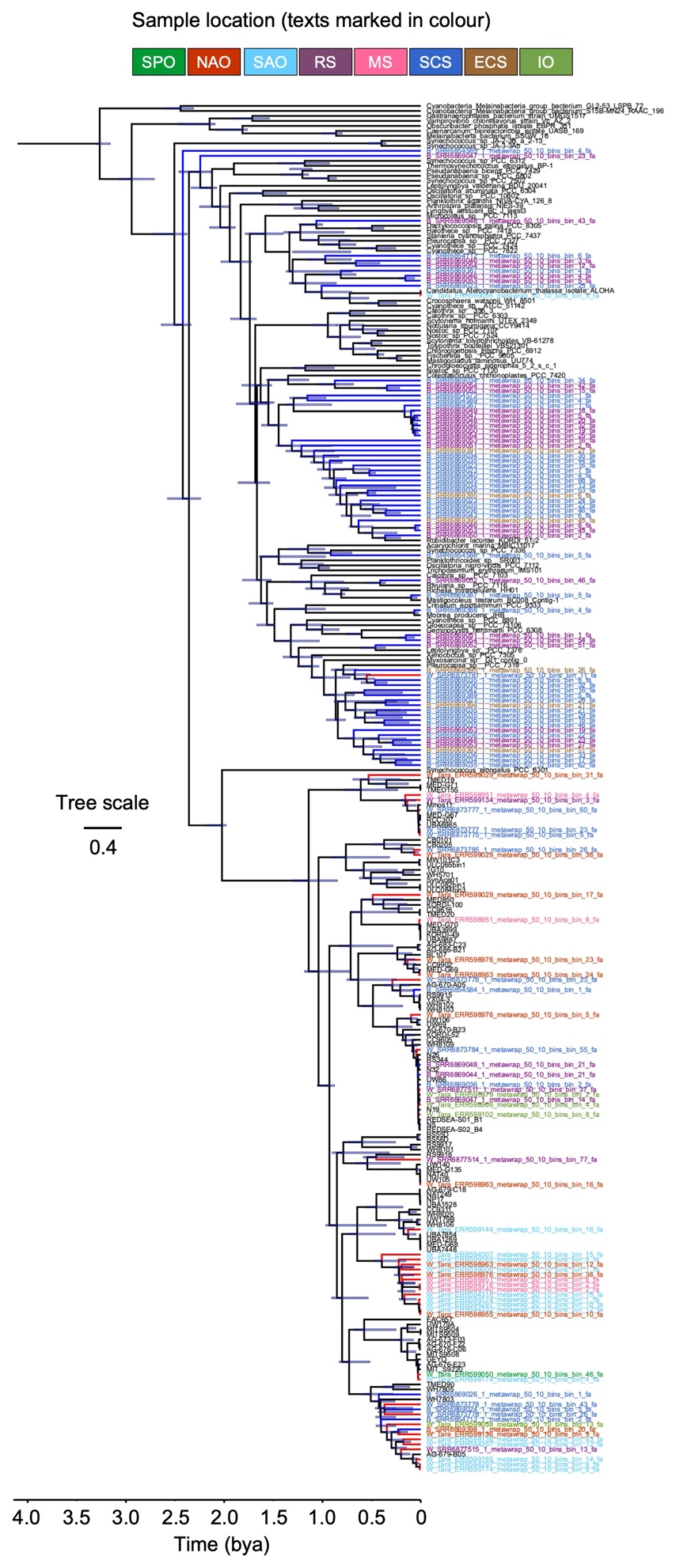


Fig. S16 Molecular dated phylogenomic tree using MAGs derived from 91 seawater (branches with red colour) and 101 biofilm (branches with blue colour) samples with 60 reference genomes. Each MAG name and its sample source location (texts marked in colours) is presented. The 95% confident interval (blue horizontal lines) of the divergent time are presented.

Fig. S17 Molecular o’clock phylogenetic tree using full-length 16S rRNA (clustered at 97% identity) derived from global-wide seawater and biofilm samples, and reference genomes that represent each subsection of distinct morphology. The posterior values are presented on branches. The blue horizontal lines are 95% confident intervals for divergent time.

Fig. S18 Molecular o’clock phylogenetic tree using full-length 16S rRNA (clustered at 99% identity) derived from global-wide seawater and biofilm samples, and reference genomes that represent each subsection of distinct morphology. The posterior values are presented on branches. The blue horizontal lines are 95% confident intervals for divergent time.

Table S1.

Alpha-diversity measurement.

| Type | Observed OTU | Chao1 | ACE | Inverse Simpson | Shannon |
| --- | --- | --- | --- | --- | --- |
| Biofilm | 42.3, 32, 33 | 65.4, 55, 41.7 | 70.8, 60.9, 38.4 | 14.1, 14.5, 6.95 | 2.88, 3.02, 0.71 |
| Seawater | 79.2, 85, 41.5 | 108, 119, 51.8 | 108, 122, 49.4 | 15.7, 16.0, 6.82 | 3.16, 3.34, 0.63 |

101 marine biofilm samples and 91 seawater samples are included for computing Observed richness, Chao1 richness, and Shannon diversity indexes. 100 marine biofilm and 91 seawater samples are included for computing ACE richness and Inverse Simpson diversity indexes, one biofilm sample with no cyanobacteria detected is omitted because unmeasurable for these two indexes. The average value and standard deviation are presented in this table (average, median, standard deviation).

**Table S2.**

Average degree, betweenness centrality, and eigenvector centrality

| Type | Average degree | Average betweenness centrality | Average eigenvector centrality |
| --- | --- | --- | --- |
| Seawater | 63 | 80 | 0.67 |
| Biofilm | 32 | 160 | 0.14 |

**References**

1. Li W, Godzik A. Cd-hit: A fast program for clustering and comparing large sets of protein or nucleotide sequences. Bioinformatics. 2006;22:1658–9.
2. Schirrmeister BE, de Vos JM, Antonelli A, Bagheri HC. Evolution of multicellularity coincided with increased diversification of cyanobacteria and the Great Oxidation Event. Proc Natl Acad Sci U S A. 2013;110:1791–6.
3. Seemann T. barrnap 0.9 : rapid ribosomal RNA prediction.
4. Katoh K, Misawa K, Kuma K, Miyata T. MAFFT: A novel method for rapid multiple sequence alignment based on fast fourier transform. Nucleic Acids Res. 2002;30:3059–66.
5. Darriba Di, Posada D, Kozlov AM, Stamatakis A, Morel B, Flouri T. ModelTest-NG: A New and Scalable Tool for the Selection of DNA and Protein Evolutionary Models. Mol Biol Evol. 2020;37:291–4.
6. Ronquist F, Huelsenbeck JP. MrBayes 3: Bayesian phylogenetic inference under mixed models. Bioinformatics. 2003;19:1572–4.
7. Rambaut A, Drummond AJ, Xie D, Baele G, Suchard MA. Posterior summarization in Bayesian phylogenetics using Tracer 1.7. Syst Biol. 2018;67:901–4.
8. Yu G, Smith DK, Zhu H, Guan Y, Lam TTY. Ggtree: an R Package for Visualization and Annotation of Phylogenetic Trees With Their Covariates and Other Associated Data. Methods Ecol Evol. 2017;8:28–36.
9. Tribble CM, Freyman WA, Landis MJ, Lim Y, Barido-Sottani J, Kopperud BT, et al. RevGadgets: an R Package for visualizing Bayesian phylogenetic analyses from RevBayes. bioRxiv. 2021;2021.05.10.443470.
10. Schirrmeister BE, Gugger M, Donoghue PCJ. Cyanobacteria and the Great Oxidation Event: Evidence from genes and fossils. Palaeontology. 2015;58:769–85.
11. Casaburi G, Duscher AA, Reid RP, Foster JS. Characterization of the stromatolite microbiome from Little Darby Island, The Bahamas using predictive and whole shotgun metagenomic analysis. Environ Microbiol. 2016;18:1452–69.
12. D’Agostino PM, Woodhouse JN, Liew HT, Sehnal L, Pickford R, Wong HL, et al. Bioinformatic, phylogenetic and chemical analysis of the UV-absorbing compounds scytonemin and mycosporine-like amino acids from the microbial mat communities of Shark Bay, Australia. Environ Microbiol. 2019;21:702–15.
13. Soo RM, Hemp J, Parks DHP, Fischer WW, Hugenholtz P. On the origin of oxygenic photosynthesis and Cyanobacteria. Science (1979). 2017;1436–40.
14. Lalonde S v., Konhauser KO. Benthic perspective on Earth’s oldest evidence for oxygenic photosynthesis. Proc Natl Acad Sci U S A. 2015;112:995–1000.
15. Robbins LJ, Lalonde S v., Planavsky NJ, Partin CA, Reinhard CT, Kendall B, et al. Trace elements at the intersection of marine biological and geochemical evolution. Earth Sci Rev [Internet]. 2016;163:323–48. Available from: http://dx.doi.org/10.1016/j.earscirev.2016.10.013
16. Chen J, Li Y, Jing H, Zhang X, Xu Z, Xu J, et al. Genomic and transcriptomic evidence for the diverse adaptations of Synechococcus subclusters 5.2 and 5.3 to mesoscale eddies. New Phytologist. 2022;233:1828–42.
17. Sanfilippo JE, Nguyen AA, Garczarek L, Karty JA, Pokhrel S, Strnat JA, et al. Interplay between differentially expressed enzymes contributes to light color acclimation in marine Synechococcus. Proc Natl Acad Sci U S A. 2019;116:6457–62.
18. Grébert T, Doré H, Partensky F, Farrant GK, Boss ES, Picheral M, et al. Light color acclimation is a key process in the global ocean distribution of Synechococcus cyanobacteria. Proc Natl Acad Sci U S A. 2018;115:E2010–9.
19. Sunagawa S, Coelho LP, Chaffron S, Kultima JR, Labadie K, Salazar G, et al. Structure and function of the global ocean microbiome. Science (1979). 2015;348:1–10.
20. Zhang W, Ding W, Li YX, Tam C, Bougouffa S, Wang R, et al. Marine biofilms constitute a bank of hidden microbial diversity and functional potential. Nat Commun. 2019;10:1–10.
